# *MdMKK9*-Mediated the Regulation of Anthocyanin Synthesis in Red-Fleshed Apple in Response to Different Nitrogen Signals

**DOI:** 10.3390/ijms23147755

**Published:** 2022-07-14

**Authors:** Xiaohong Sun, Xinxin Li, Yanbo Wang, Jihua Xu, Shenghui Jiang, Yugang Zhang

**Affiliations:** 1 Key Laboratory of Plant Biotechnology of Shandong Province, College of Life Sciences, Qingdao Agricultural University, Qingdao 266109, China; mingsun9887@163.com (X.S.); xujihua@qau.edu.cn (J.X.); 2 Engineering Laboratory of Genetic Improvement of Horticultural Crops of Shandong Province, Qingdao Agricultural University, Qingdao 266109, China; 3 College of Horticulture, Qingdao Agricultural University, Qingdao 266109, China; lixinxinll@163.com (X.L.); 15820054339@163.com (Y.W.)

**Keywords:** *MdMKK9*, nitrogen signals, red-fleshed apple, CRISPR/Cas9, anthocyanin

## Abstract

The mitogen-activated protein kinase (MAPK) signaling cascade is a widely existing signal transduction system in eukaryotes, and plays an important role in the signal transduction processes of plant cells in response to environmental stress. In this study, we screened *MdMKK9*, a gene in the MAPK family. This gene is directly related to changes in anthocyanin synthesis in the ‘Daihong’ variety of red-fleshed apple (*Malus sieversii f neidzwetzkyana* (Dieck) Langenf). *MdMKK9* expression was up-regulated in ‘Daihong’ tissue culture seedlings cultured at low levels of nitrogen. This change in gene expression up-regulated the expression of genes related to anthocyanin synthesis and nitrogen transport, thus promoting anthocyanin synthesis and causing the tissue culture seedlings to appear red in color. To elucidate the function of *MdMKK9*, we used the CRISPR/Cas9 system to construct a gene editing vector for *MdMKK9* and successfully introduced it into the calli of the ‘Orin’ apple. The *MdMKK9* deletion mutants (MUT) calli could not respond to the low level of nitrogen signal, the expression level of anthocyanin synthesis-related genes was down-regulated, and the anthocyanin content was lower than that of the wild type (WT). In contrast, the *MdMKK9*-overexpressed calli up-regulated the expression level of anthocyanin synthesis-related genes and increased anthocyanin content, and appeared red in conditions of low level of nitrogen or nitrogen deficiency. These results show that *MdMKK9* plays a role in the adaptation of red-fleshed apple to low levels of nitrogen by regulating the nitrogen status and anthocyanin accumulation.

## 1. Introduction

Red-fleshed apple (*Malus sieversii f neidzwetzkyana* (Dieck) Langenf) is rich in anthocyanins and other flavonoids that have anti-oxidation and anti-radiation effects and help prevent cardiovascular and cerebrovascular diseases and arteriosclerosis [1,2]. At present, red-fleshed apple is cultivated in various countries, including New Zealand [3] and Can-ada [4]. In 2017, a new red-fleshed apple variety ‘Daihong’ (variety right number: CNA20162427.9) with the R1R6 genotype was bred at Qingdao Agricultural University. ‘Daihong’ fruits are sour and crispy, and can be used as fresh food and to produce processed juices [5]. In addition, this variety contains higher levels of anthocyanin than white-fleshed apples.

Anthocyanins are a major health-promoting component of red-fleshed apples. To date, studies on the biosynthesis of anthocyanins have mainly focused on their structure genes, including chalcone synthase (*CHS*), chalcone isomerase (*CHI*), flavanone 3-hydroxylase (*F3H*), dihydroflavonol 4-reductase (*DFR*), anthocyanidin synthase (*ANS*), uridine diphosphate-glucose: flavonoid-O-glycosyltransferase (*UFGT*) and anthocyanidin reductase (*ANR*), and regulatory genes, including myeloblastosis (*MYB*), helix-loop-helix (*bHLH*) and WD40-repeat (*WD40*) [3,6,7,8,9,10,11]. Anthocyanin biosynthesis is regulated by a variety of internal and external factors, including cultivation conditions, plant hormone levels, non-hormone chemicals, and genetic regulation [12,13]. The main external environmental conditions that are important for anthocyanin biosynthesis are light, temperature, and nitrogen levels [14,15]. The nitrate uptake by the root from the soil and movement of nitrate inside the plant is mediated by the membrane-bound nitrate transporters. In plants, three multigenic families (NPF, NRT2 and AMT) are involved in N uptake and utilization [16,17]. 17 *NPF6* genes were identified in apples [18], these apple *MdNPFs* are structurally conserved, based on alignment of amino acid sequences and analyses of phylogenetics and conserved domains [19]. Wang et al. reported that overexpressing *MdNPF6.5* in apple calli improved the tolerance to low nitrogen stress and the nitrogen absorption capacity [19]. Huang et al. reported that two NRT family gene *MdNRT2.4* and *MdNRT2.7* were involved in the response to PEG induced drought stress in apple [20]. Expression analysis of red-fleshed apples revealed that nitrate starvation induced the expression of *MdNRT2.4* [21]. Overexpressing the high-affinity transporter *MdNRT2.4* enhanced low N stress tolerance in *Arabidopsis* [22]. When the *MdTyDc* was overexpressed in apple leaves, the transcript levels of N-absorption-related genes (i.e., *MdAMT1.5*, *MdAMT3.1*) were higher than in WT lines under alkaline stress [23]. The expression of *MdAMT3.1* in apple roots increased significantly with the decrease of N supply level [20]. Nitrogen deficiency has been found to significantly promote anthocyanin accumulation in *Arabidopsis*, grape, and radish [6,24,25]. High nitrogen concentrations induce transcription factors such as LBD37, LBD38, and LBD39—members of the lateral organ boundary domain (LBD) protein family in *Arabidopsis*—that negatively regulate anthocyanin synthesis by inhibiting the expression of genes related to anthocyanin synthesis [6]. In *Arabidopsis thaliana*, the DELLA protein (whose N-terminal has a conserved amino acid sequence, D-E-L-L-A) can directly interact with PAP1 to regulate nitrogen stress-induced anthocyanin accumulation [26]. The BTB/TAZ protein (encoded by the nitrate response gene *MdBT2*) interacts with *MdMYB1* to regulate anthocyanin accumulation in apple [8]. Moreover, the overexpression of *MdATG18a* can improve anthocyanin accumulation in transgenic apple lines and enhance their tolerance to low nitrogen stress [27].

The mitogen-activated protein kinase (MAPK) signaling cascade is a signal transduction system that is widely distributed in eukaryotes and plays an important role in the signal transduction processes of plant cells responding to environmental stress [28,29,30]. MAPK cascades are key signaling modules downstream of receptors or sensors that perceive endogenous and exogenous stimuli, such as hormones, peptide ligands, and effectors [31]. For example, *MdMKKK1* regulates resistance to *Botryosphaeria dothidea* infection in apples [32], and *MKK9* negatively regulates abiotic stress response and enhances abscisic acid levels and salt tolerance in *Arabidopsis* [33]. In *Arabidopsis*, MKK9 is an important member of group D MKKs (a MAPK signaling cascade pathway) and regulates anthocyanin synthesis in response to low nitrogen levels and phosphorus stress [34,35]. The MKK9–MPK6 cascade plays a role in the salt stress response of A. thaliana by regulating the phosphorylation of RCA, FTSZ2-2, TOR2, and PRPS1 [36]. MKK9 is a positive factor for H_2_O_2_ accumulation in salt-treated calli of *A. thaliana*, and plays a key role in enhancing respiration by forming the cascade with MAPK3/6 [37]. MPK6 is a direct target of MKK9, and its knockout delays leaf senescence [38]. *MKK9* also regulates the adaptation of *Arabidopsis* plants to low nitrogen stress by regulating their anthocyanin levels and nitrogen status [39]. Although the genome-wide analysis of the MAPK family has been carried out in some species, little is known about *MAPKK* genes in apple [29]. Moreover, it is not clear how *MKK9* regulates anthocyanin synthesis in apple under nitrogen stress.

The excessive use of nitrogen in apple orchards reduces fruit coloring and quality, which affects their commercial value and edibility [40,41]. In this study, we investigated how *MdMKK9* regulates anthocyanin accumulation in apple in response to low nitrogen stress. Our results provide a basis for the rational application of nitrogen fertilizers in the production and cultivation of new red-fleshed apple varieties.

## 2. Results

### 2.1. MdMKK9 Is Directly Related to Changes in the Anthocyanin Content of Red-Fleshed Apples

**Figure 1:**
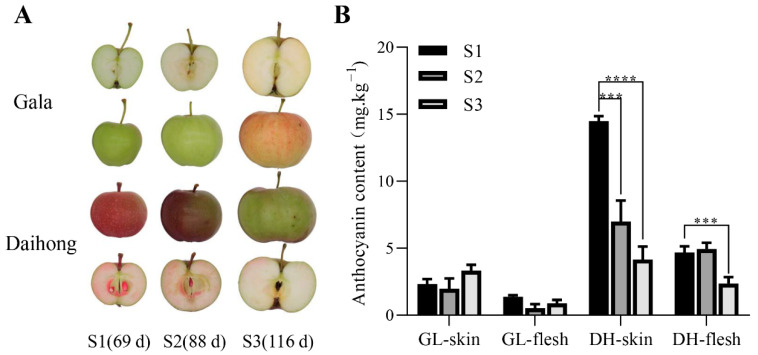
The phenotypes and anthocyanin contents of ‘Gala’ and ‘Daihong’ apples at different stages of growth. (**A**): Color changes in the skin and flesh of ‘Gala’ (GL) and ‘Daihong’ (DH) apples during the S1, S2, and S3 stages of fruit development. (**B**): Anthocyanin contents of the skin and flesh of ‘Gala’ and ‘Daihong’ apples during the S1, S2, and S3 stages. The bars represent means ± SD (*n* = 3). Asterisks indicate statistically significant differences (*** *p* < 0.001; **** *p* < 0.0001).

‘Daihong’ fruits were collected for observation at 69 d (S1), 88 d (S2), and 116 d (S3) after full bloom (Figure 1A). Corresponding samples were collected from ‘Gala’ (white-fleshed apple) plants as a control. ‘Daihong’ S1 (young fruits at an early growth stage) had red skin, pink flesh, and high levels of anthocyanin. With increasing fruit volume at stages S2 and S3, the color of the skin and flesh became lighter and the anthocyanin levels decreased. However, anthocyanin levels were higher in ‘Daihong’ than in ‘Gala’ at all stages of growth (Figure 1B).

**Figure 2:**
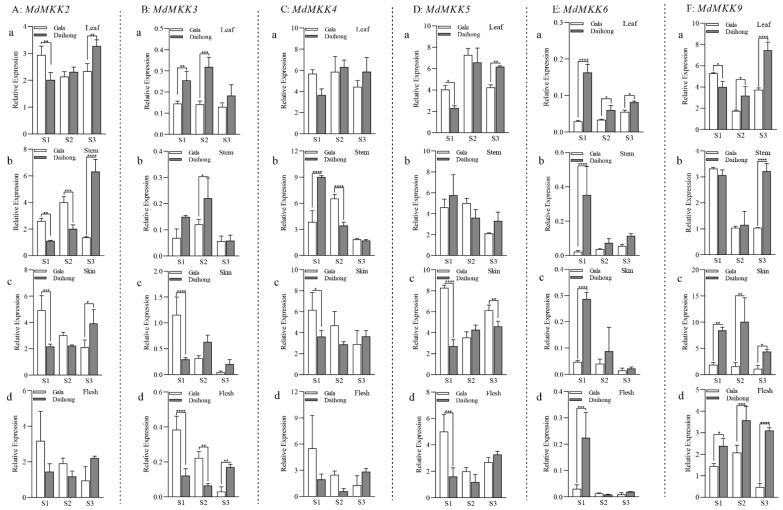
Relative expression levels of genes in the MAPK gene family at different developmental stages in ‘Gala’ and ‘Daihong’. (**A**) *MdMKK2* (**a**, Leaf, **b**, Stem, **c**, Skin, **d**, Flesh, the same below). (**B**) *MdMKK3*. (**C**) *MdMKK4*. (**D**) *MdMKK5*. (**E**): *MdMKK6*. (**F**): *MdMKK9*. The relative gene expression was calculated according to the 2^−ΔCT^ method. The bars represent means ± SD (*n* = 3). Asterisks indicate statistically significant differences (* *p* < 0.05; ** *p* < 0.01; *** *p* < 0.001; **** *p* < 0.0001).

The MAPK family of genes plays an important role in the growth, development, and stress resistance of A. thaliana. We obtained the *MdMKK2–MdMKK9* sequences from NCBI and designed primers to measure the expression levels of these genes in the leaves, stems, skin, and flesh of ‘Daihong’ and ‘Gala’ fruits at different developmental stages (Figure 2). The expression levels of *MdMKK2* were higher in the leaves, stems, skin, and flesh of ‘Gala’ than in those of ‘Daihong’ in the S1 and S2 stages (Figure 2A,B). *MdMKK2* expression was highest in the stems of ‘Daihong’ in the S3 stage (Figure 2C). The expression levels of *MdMKK3* were higher in leaves and stems of ‘Daihong’ than in those of ‘Gala’ in all stages, and higher in the flesh of ‘Daihong’ than in that of ‘Gala’ only in S3. *MdMKK4* expression was higher in the skin and flesh of ‘Daihong’ than in those of ‘Gala’ in S3. *MdMKK5* expression was higher in the leaves and stems of ‘Daihong’ than in those of ‘Gala’ in S3; however, its expression level in the fruit flesh was always lower in ‘Daihong’ than in ‘Gala’. The expression level of *MdMKK6* was higher in the leaves and stems of ‘Daihong’ than in those of ‘Gala’; however, its expression level in the fruit flesh was lower in ‘Daihong’ than in ‘Gala’ in the S2 stage. The expression levels of *MdMKK9* in the leaves and stems of ‘Daihong’ increased gradually relative to those in the leaves and stems of ‘Gala’ from S1 to S3. *MdMKK9* expression was higher in the skin and flesh of ‘Daihong’ than in those of ‘Gala’, and decreased gradually from S1 to S3. The above results suggested that only the expression levels of *MdMKK9* gene in both skin and flesh of red-fleshed apple were significantly higher than that in white-fleshed apples at the three developmental stages. We speculated that the expression level of *MdMKK9* was closely related to changes in the anthocyanin contents of the fruits, and *MdMKK9* was thus selected for further study in subsequent experiments.

### 2.2. The Increase in Anthocyanin Content Is Mediated by the Up-Regulation of MdMKK9 in ‘Daihong’ under Low Nitrogen Stress

**Figure 3:**
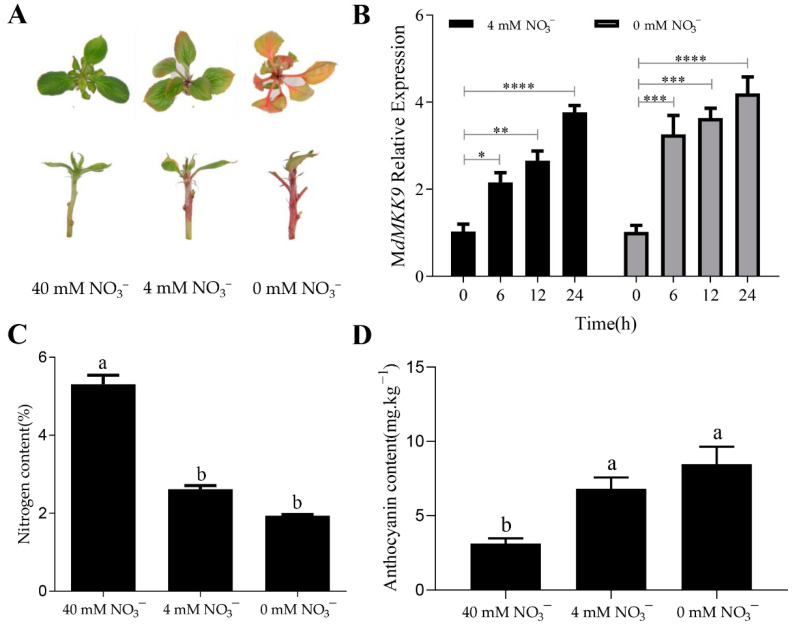
The phenotype, nitrogen content, anthocyanin content, and relative expression level of the *MdMKK9* gene in ‘Daihong’ tissue culture seedlings grown in culture mediums with different nitrogen concentrations. (**A**) Phenotype. (**B**) Relative expression levels of *MdMKK9*. The relative level of *MdMKK9* in ‘Dahong’ tissue culture seedlings at 0 h was set to 1. Asterisks indicate statistically significant differences (* *p* < 0.05; ** *p* < 0.01; *** *p* < 0.001; **** *p* < 0.0001). (**C**) Nitrogen content. (**D**) Anthocyanin content. In (**C**,**D**), different letters above the bars indicate significant differences (*p* < 0.05). The bars represent means ± SD (*n* = 3).

In tissue culture seedlings grown on nitrogen-deficient mediums (0 and 4 mM NO_3_^−^), the leaves and stems gradually turned red with increasing culture duration; the lower the nitrogen concentration, the darker the red color (Figure 3A). The relative expression of *MdMKK9* was measured by qRT-PCR at various time points across 24 h of culture. With increasing culture duration, the relative expression levels of *MdMKK9* increased significantly in tissue culture seedlings grown on mediums with 4 mM and 0 mM NO_3_^−^. The lower the nitrogen concentration, the higher the expression of *MdMKK9* (Figure 3B). The ‘Daihong’ tissue culture seedlings were further grown in the different mediums for 25 days, and the nitrogen content of the tissues were measured again. The nitrogen content of plant tissues decreased with decreasing nitrogen concentration of the medium (Figure 3C). There was no significant difference in the nitrogen content of tissue culture seedlings grown on culture mediums with 4 mM and 0 mM NO_3_^−^. However, there was a significant difference in nitrogen content between tissue culture seedlings grown with 40 mM NO_3_^−^ and those grown with 4 mM and 0 mM NO_3_^−^ (Figure 3C). The anthocyanin content of ‘Daihong’ tissue culture seedlings increased with decreasing nitrogen concentration of the medium (Figure 3D). There was no significant difference in anthocyanin content between tissue culture seedlings grown at nitrogen concentrations of 4 mM and 0 mM NO_3_^−^, both of which had significantly higher anthocyanin levels than the tissue culture seedlings grown at 40 mM NO_3_^−^ (Figure 3D). Therefore, we speculated that *MdMKK9* was involved in increasing the anthocyanin content of ‘Daihong’ tissue culture seedlings under low nitrogen stress. Moreover, the application of a small amount of nitrogen to the growth medium (40 mM/4 mM = 10X) did not decrease nitrogen uptake by plants to the same extent (5.3/2.3 = 2.3X).

**Figure 4:**
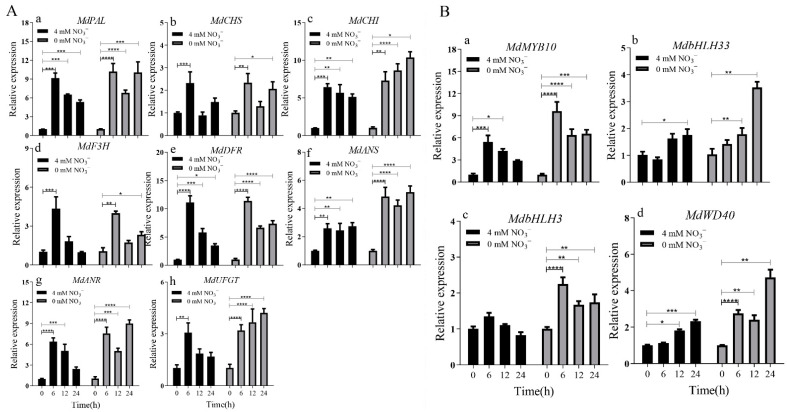
Relative expression of anthocyanin synthesis-related genes in ‘Daihong’ tissue culture seedlings grown in mediums with 4 and 0 mM NO_3_^−^. (**A**) Relative expression of structural genes involved in anthocyanin synthesis (**a**: *MdPAL,* **b***: MdCHS,* **c***: MdCHI,* **d**: *MdF3H*, **e***: MdDFR*, **f***: MdANS,* **g***: MdANR,* **h***: MdUFGT*). (**B**) Relative expression of regulatory genes related to anthocyanin synthesis (**a**: *MdMYB10*, **b**: *MdbHLH33*, **c**: *MdbHLH3*, **d**: *MdWD40*). The relative level of anthocyanin synthesis-related genes in ‘Dahong’ tissue culture seedlings at 0 h was set to 1. The bars represent means ± SD (*n* = 3). Asterisks indicate statistically significant differences (* *p* < 0.05; ** *p* < 0.01; *** *p* < 0.001; **** *p* < 0.0001).

In this case, qRT-PCR was used to detect the relative expression level of structural genes related to anthocyanin synthesis—including *MdPAL*, *MdCHS*, *MdCHI*, *MdF3H*, *MdDFR*, *MdANS*, *MdANR* and *MdUFGT*—in tissue culture seedlings grown in mediums with 4 and 0 mM NO_3_^−^ for 24 h. The time taken to reach the maximum expression level differed between genes and culture mediums (Figure 4). The expression levels of all genes were highest at 6 h in the 4 mM NO_3_^−^ medium (Figure 4A), whereas the expression levels of *MdCHI*, *MdANR* and *MdUFGT* were highest at 24 h. In the 0 mM NO_3_^−^ medium, the expression levels of *MdCHS*, *MdF3H* and *MdDFR* were highest at 6 h in the 0 mM NO_3_^−^ medium. The expression levels of regulatory genes (*MYB10*, *MdbHLH3*, *MdbHLH33*, and *MdWD40*) were higher in the 0 mM NO_3_^−^ medium than in the 4 mM NO_3_^−^ medium. The expression levels of *MdMYB10* and *MdbHLH3* were highest at 6 h in both mediums, whereas those of *MdbHLH33* and *MdWD40* were highest at 24 h (Figure 4B).

**Figure 5:**
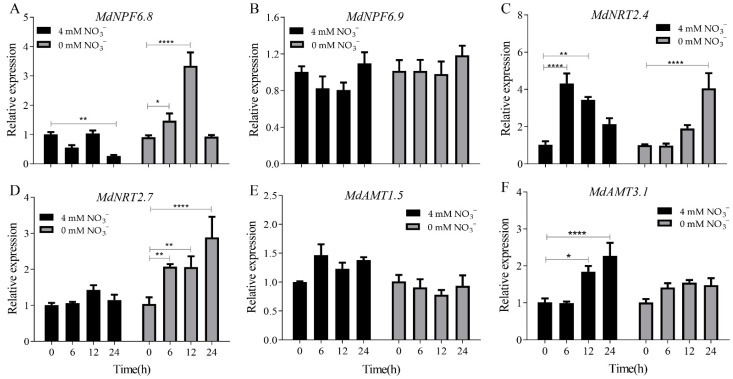
Relative expression of nitrogen transport-related genes in tissue cultured seedlings of ‘Daihong’ cultured in mediums with 4 and 0 mM NO_3_^−^. (**A**). *MdNPF6.8*, (**B**). *MdNPF*6.9, (**C**). *MdNRT2.4*, (**D**) *MdNRT2.7*, (**E**) *MdAMT1.5,* (**F**) *MdNMT3.1.* The relative level of nitrogen transport-related genes in ‘Dahong’ tissue culture seedlings at 0 h was set to 1. The bars represent means ± SD (*n* = 3). Asterisks indicate statistically significant differences (* *p* < 0.05; ** *p* < 0.01; **** *p* < 0.0001).

Most studies on nitrogen uptake and transport in plants focus on *A. thaliana*, a model plant. For our experiments, we selected the following apple genes: *MdNFP6.8–6.9*, *MdNRT2.4–2.7* and *MdAMT1.5–3.1.* MdNFP6.8–6.9 and AtNRT1.1 have high amino acid sequence homology (57.1−45.3%); MdNRT2.4–2.7 had high homology with AtNRT2.1 family in amino acid sequence (71.3−75.6%); and *MdAMT1.5–3.1* from the ammonium transporter gene family of apple (The homology of amino acid sequence between MdAMT1.5–3.1 and other plant AMTs were shown in Supplementary Figure S1). We obtained the relative expression of nitrogen transport-related genes in tissue cultured seedlings of ‘Daihong’ cultured in mediums with 4 and 0 mM NO_3_^−^ (Figure 5). The expression level of *MdNFP6.8* (Figure 5A) was highest at 12 h in the 0 mM NO_3_^−^ medium, whereas those of *MdNFP6.9* (Figure 5B) had changed little in the 4 and 0 mM NO_3_^−^ medium. The expression level of *MdNRT2.4* (Figure 5C) at 6 h was highest in the 4 mM NO_3_^−^ medium, and then gradually decreased, the expression level of *MdNRT2.4* increased gradually in the 0 mM NO_3_^−^ medium and reached the highest level in 24 h. The expression levels of *MdNRT2.7* (Figure 5D) were higher in the 0 mM NO_3_^−^ medium than in the 4 mM NO_3_^−^ medium, whereas *MdAMT1.5* and *MdAMT3.1* (Figure 5E,F) showed the opposite trend. Among these above genes, only the expression level of *MdNRT2.7* in 0 mM NO_3_^−^ medium was always higher than that in 4 mM NO_3_^−^ medium, and increased with the extension of treatment time.

### 2.3. Calli with MdMKK9 Deletion No Longer Respond to the Low Nitrogen Signal

#### 2.3.1. Construction of a CRISPR/Cas9 *MdMKK9* Gene Editing Vector and Obtaining *MdMKK9* Deletion Mutant Calli

To examine whether *MdMKK9* could increase the anthocyanin content of apple in response to a low nitrogen signal, we cloned the coding sequence region of *MdMKK9* (GenBank accession number: ON427820) from the ‘Daihong’ variety of red-fleshed apple, constructed an overexpression vector, and successfully introduced it into the calli of the ‘Orin’ variety. We obtained six *MdMKK9* overexpressed calli lines (OE) in which the relative expression levels of *MdMKK9* were significantly higher than those in wild-type calli (WT) (Supplementary Figure S2).

**Figure 6:**
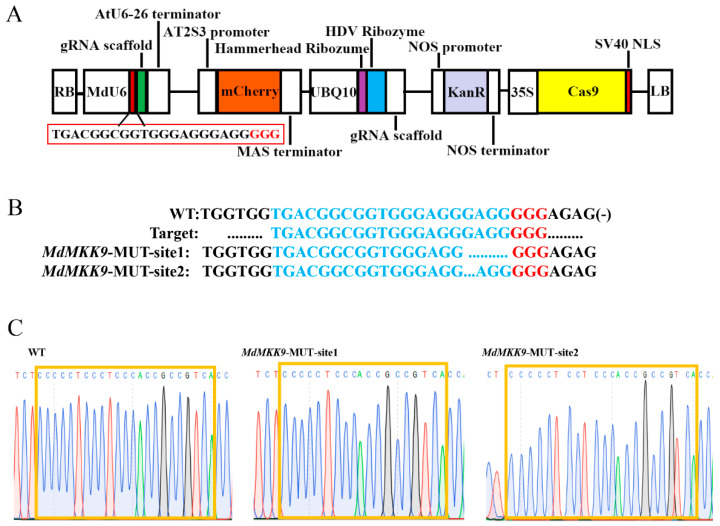
Mutagenesis of the *MdMKK9* gene based on the CRISPR/Cas9 system. (**A**) Expression cassette of the gene-editing vector derived from pHDE-35S-Cas9-mCherry-UBQ. The red box indicates the target site. (**B**) *MdMKK9* mutations resulting from four positive lines of gene editing. WT, wild-type. *MdMKK9*-MUT-site1 and *MdMKK9*-MUT-site2 represent two different clones. The blue dots indicate the missing bases. Red letters indicate the protospacer adjacent motifs. (**C**) Sequencing chromatogram of partial genomic DNA of *MdMKK9* corresponding to the mutations. The orange frames indicate target sites for gene editing.

To elucidate the function of the *MdMKK9* gene, we performed gene editing in apple calli based on the CRISPR/Cas9 system to produce mutagenesis in the *MdMKK9* gene. We selected target sequences from the exons of the *MdMKK9* gene using the online CCTOP tool and constructed a gene editing vector (Figure 6A). The new construct was introduced into ‘Orin’ calli using Agrobacterium-mediated transformation methods obtaining *MdMKK9* deletion mutant calli (MUT). Kanamycin-resistant calli were subcultured twice, and calli that tested positive for transformation were subjected to molecular examination. We sequenced four positive calli lines and found two different sequence mutations at the target site in all sequenced calli. The mutations included a 4-bp deletion at *MdMKK9*-MUT-site1 and a 1-bp deletion at *MdMKK9*-MUT-site2 (Figure 6B,C).

#### 2.3.2. Increase in the Anthocyanin Content of Calli in Transgenic ‘Orin’ under Low Nitrogen Stress Is Mediated by the Up-Regulation of *MdMKK9*

**Figure 7:**
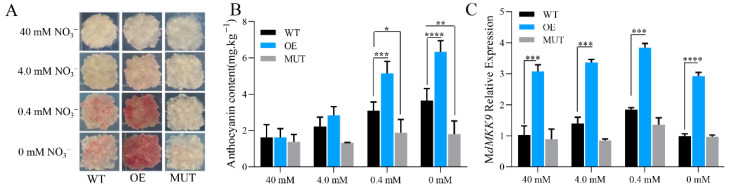
The phenotype, anthocyanin content, and *MdMKK9* expression levels of calli from wild-type (WT) ‘Orin’ plants, transformed CRISPR/Cas9-*MdMKK9* (MUT) lines, and transformed pRI101-*MdMKK9* (OE) lines grown in mediums with different nitrogen concentrations. (**A**) Phenotypes of the calli. (**B**) Anthocyanin content. (**C**) Relative expression levels of *MdMKK9*. The relative level of *MdMKK9* in 40 mM NO_3_^−^ treated WT calli was set to 1. The bars represent means ± SD (*n* = 3). Asterisks indicate statistically significant differences (* *p* < 0.05; ** *p* < 0.01; *** *p* < 0.001; **** *p* < 0.0001).

The calli of WT, OE, and MUT lines were cultured on mediums with 40, 4, 0.4, and 0 mM NO_3_^−^ for 5 d at 16 °C under dark conditions. The OE calli began to appear slightly red at 4 mM NO_3_^−^, and the red phenotype gradually deepened with decreasing nitrogen concentration of the medium. The WT calli started to appear red at 0.4 mM NO_3_^−^, and the MUT lines exhibited a red phenotype at 0 mM NO_3_^−^. The anthocyanin contents of WT and OE calli increased with decreasing nitrogen concentration the medium, and there were significant differences within and across culture conditions. The anthocyanin contents of MUT calli did not differ significantly between culture conditions with different nitrogen concentration (Figure 7B). The relative expression levels of the *MdMKK9* gene are shown in Figure 7C. In all four nitrogen concentrations, the expression levels of *MdMKK9* were significantly higher in OE calli than in WT calli and MUT calli. However, there were no significant differences between WT calli and MUT calli.

**Figure 8:**
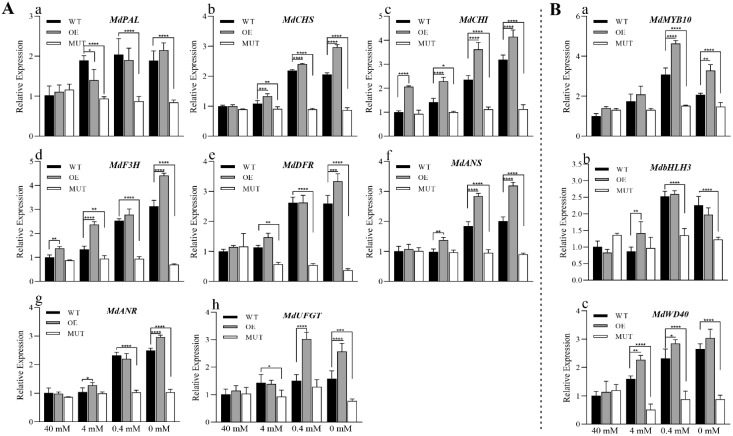
Expression levels of structural genes related to anthocyanin synthesis in the calli of wild-type (WT) plants, transformed CRISPR/Cas9-*MdMKK9* (MUT) lines, and transformed pRI101-*MdMKK9* (OE) lines grown in mediums with different nitrogen concentrations. (**A**) Relative expression of structural genes involved in anthocyanin synthesis (**a**: *MdPAL*, **b**: *MdCHS*, **c**: *MdCHI*, **d**: *MdF3H*, **e**: *MdDFR*, **f**: *MdANS*, **g**: *MdANR*, **h**: *MdUFGT*). (**B**) Relative expression of regulatory genes related to anthocyanin synthesis (**a**: *MdMYB10*, **b**: *MdbHLH3*, **c**: *MdWD40*). The relative level of anthocyanin synthesis-related genes in 40 mM NO_3_^−^ treated WT calli was set to 1. The bars represent means ± SD (*n* = 3). Asterisks indicate statistically significant differences (* *p* < 0.05; ** *p* < 0.01; *** *p* < 0.001; **** *p* < 0.0001).

In this case, qRT-PCR was used to determine the relative expression levels of anthocyanin synthesis-related structural genes (*MdPAL*, *MdCHS*, *MdCHI*, *MdF3H*, *MdDFR*, *MdANS*, *MdANR* and *MdUFGT*) (Figure 8A) and regulatory genes (*MdMYB10*, *MdbHLH3* and *MdWD40*) (Figure 8B) in the calli of different lines. In growth mediums with 40, 4, 0.4 and 0 mM NO_3_^−^, there were progressively increased in the expression levels of all anthocyanin synthesis-related genes in WT and OE calli. The expression levels of those genes in MUT calli showed little difference across growth mediums with different nitrogen concentrations. In the culture medium with 0.4 mM NO_3_^−^, the expression level of each gene increased significantly in the OE and WT lines and remained high even with decreasing nitrogen concentrations of the medium. In culture mediums with 0.4 mM and 0 mM NO_3_^−^, the gene expression levels of OE lines were slightly higher than those of WT lines and significantly higher than those of MUT lines.

**Figure 9:**
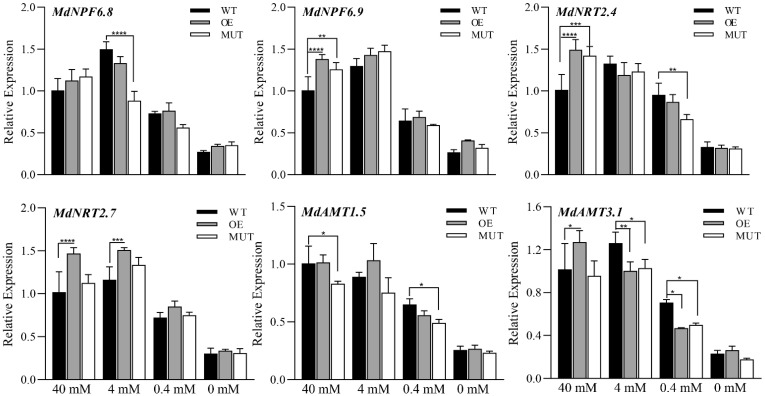
Expression levels of nitrogen regulatory genes in the calli of wild-type (WT) plants, transformed CRISPR/Cas9-*MdMKK9* (MUT) lines, and transformed pRI101-*MdMKK9* (OE) lines grown in mediums with different nitrogen concentrations. The relative level of nitrogen transport-related genes in 40 mM NO_3_^−^ treated WT calli was set to 1. The bars represent means ± SD (*n* = 3). Asterisks indicate statistically significant differences (* *p* < 0.05; ** *p* < 0.01; *** *p* < 0.001; **** *p* < 0.0001).

Overall, the expression levels of nitrogen transport-related genes in WT, OE and MUT calli decreased with decreasing nitrogen concentration of the medium (Figure 9). The relative expression levels of *MdNFP6.9*, *MdNRT2.4*, and *MdAMT3.1* in OE calli cultured at 40 mM NO_3_^−^ were higher than those of MUT and WT calli. The relative expression levels of *MdNFP6.8* and *MdAMT1.5* in OE calli were not significantly different from those in MUT and WT, and there were no differences in color between these calli at this time. The expression of nitrogen transport-related genes in different calli in the 0 mM NO_3_^−^ treatment did not change significantly, however, the relative expression levels of *MdNPF6.9*, *MdNTR2.7* and *MdAMT3.1* in OE calli were higher than those in MUT and WT calli. The expression levels of genes in the 0.4 mM and 0 mM NO_3_^−^ treatments were slightly lower compared to those in the 40 mM and 4 mM NO_3_^−^ treatments, respectively; however, this decrease was smaller than the corresponding decrease in the nitrogen concentration of the medium. Among all these above genes, after treated with different nitrogen concentrations, only the expression level of *MdNTR2.7* in OE calli was higher than that of WT and MUT calli.

## 3. Discussion

MAPK/MPK is a type of serine (Ser)/threonine (Thr) protein kinase that is ubiquitous in eukaryotes, including yeast, animals, and plants [42,43]. The MAPK-mediated signaling cascade is one of the most important pathways in the signal transmission network of eukaryotes. It is involved in intracellular signaling and extracellular signal amplification, induces appropriate physiological and biochemical responses in recipient cells, and plays an important role in the regulation of plant immunity [44]. MAPKKs are involved in various aspects of physiological and developmental processes in apple [29]. In this study, we identified *MdMKK9*, a member of the MAPK gene family that is closely associated with the anthocyanin content of the ‘Daihong’ variety of red-fleshed apples.

‘Daihong’ is a newly bred red-fleshed apple variety that can be used as fresh food or for processing [5]. As such, it has not been studied in detail. The anthocyanin content of the skin and flesh of ‘Daihong’ fruits decreased with fruit development, and the expression levels of the *MdMKK9* gene also decreased significantly. This indicated that *MdMKK9* is a member of the *MAPK* gene family and is directly associated with changes in the anthocyanin content of red-fleshed apples. *MdMKK9* and AtMKK9 are members of the same subfamily, and share a close genetic relationship [45] (Supplementary Figure S3). The 3D structures of *Arabidopsis* AtMKK9 and apple *MdMKK9* were very similar and share a high degree of overlap (Supplementary Figure S4). Therefore, the function of *MdMKK9* can be inferred based on the predicted functional network diagram of AtMKK9-interacting proteins.

Low nitrogen stress has been reported to induce anthocyanin accumulation in apple [9], radish [11], and grape [6]. In this study, we found that after 25 days of growth in a low-nitrogen medium, the tissue culture seedlings of ‘Daihong’ gradually turned red in color. Moreover, their anthocyanin content gradually increased with decreasing nitrogen content of the medium. Structural genes in the anthocyanin synthesis pathway (such as *MdCHS*, *MdCHI, MdDFR*, *MdANS*, and *MdUFGT*) were up-regulated in ‘Daihong’ seedlings after 24 h of treatment in low nitrogen and nitrogen-deficient conditions. Among them, MdDFR and MdUFGT are important enzymes for the increase of anthocyanin content [46]. *NRT2* is a high-affinity nitrate transporter [19], and in *A. thaliana*, *AtNRT2.7* is known to play a specific role in seed nitrate accumulation. *AtNRT2.7* is induced by nitrate and hormones, enhances the growth of the root system, and improves the absorption and utilization of nitrogen [47]. In apples, the expression of *MdNRT1.5*/*MdNPF7.3* inhibits the transport of nitrate from root to branch [48]. Nitrate has been reported to mediate phosphorus absorption and starvation signals by activating the *NIGT1-SPX-PHR* cascade signaling pathway [49]. *MdNPF6.5* confers a high capacity for nitrogen uptake under low-nitrogen conditions [19]. *AtNRT2.4* is a nitrate transporter, which can promote root uptake of nitrogen under N starvation [18]. In this study, we found that genes related to nitrogen and ammonium root transport (such as *MdNPF6.9*, *MdNRT2.4*, and *MdNRT2.7*) were up-regulated after 24 h of nitrogen treatment. Only *MdNRT2.7* expression levels were higher in conditions of nitrogen deficiency (0 mM NO_3_^−^) than under low nitrogen stress (4 mM NO_3_^−^). Moreover, the expression levels of *MdAMT1.5* and *MdAMT3.1* were higher under low nitrogen stress (4 mM NO_3_^−^) than in conditions of nitrogen deficiency (0 mM NO_3_^−^). When *MdMKK9* was normally expressed, the decrease in nitrogen content and increase in anthocyanin content in two culture conditions (4 and 0 mM NO_3_^−^) were significantly different from those in the 40 mM NO_3_^−^ culture condition. Interestingly, there were no significant differences in the nitrogen or anthocyanin contents of tissue culture seedlings between the 4 and 0 mM NO_3_^−^ growth mediums, this was suggested that plants did not significantly reduced nitrogen uptake due to low nitrogen culture.

Since it is difficult and time-consuming to obtain transgenic apple plants, we used the calli of the ‘Orin’ variety of apple plants for the functional analysis of *MdMKK9*. The calli of ‘Orin’ have some favorable traits, such as an unlimited proliferation potential, non-differentiation during subculturing, and ease of transformation by *Agrobacterium*-mediated methods. As such, it has been successfully used to study gene functions in apple [50,51,52]. The CRISPR/Cas9 system is a revolutionary genome editing technique that has been widely used in numerous plants, including *Arabidopsis* [53], citrus [54], tomato [55,56], and apple [32,52,57]. To investigate the function of the *MdMKK9*, we established gene deletion systems. Through genetic transformation and antibiotic screening, we obtained four positive clones. The sequencing results showed that the *MdMKK9* gene in the mutant ‘Orin’ calli had two mutations—a 4-bp deletion and a 1-bp deletion—at the target site that would destroy the translation of the *MdMKK9* protein. Therefore, the mutant calli were used to investigate the function of the *MdMKK9* gene. Low nitrogen is known to stimulate anthocyanin production in the skin cells of red varieties of grapes [6]; however, the color and anthocyanin content of the *MdMKK9* deletion mutant calli did not change significantly under low nitrogen stress. Thus, we speculated that the mutation of *MdMKK9* inhibited the low nitrogen-induced synthesis of anthocyanin. The accumulation patterns of certain pigments give fruits different colors, and red cultivars are generally characterized by higher levels of anthocyanins [58]. When transgenic calli with *MdMKK9* overexpression were subjected to different levels of nitrogen treatment, the calli gradually turned red, and the anthocyanin content of the red calli increased with decreasing nitrogen levels.

## 4. Materials and Methods

### 4.1. Plant Materials

The plant materials used in this study included the leaves, stems, skin, and flesh of the ‘Daihong’ and ‘Gala’ cultivars of apple, tissue culture seedlings of ‘Daihong’, the calli of the ‘Orin’ cultivar, and *Nicotiana benthamiana* leaves. ‘Daihong’ and ‘Gala’ plants at the Jiaozhou Modern Agriculture Demonstration Park of Qingdao Agricultural University were sampled at different growth stages. The calli of ‘Orin’ used in the present study were kindly provided by Prof. Songling Bai from the College of Agriculture and Biotechnology, Zhejiang University, Hangzhou, China.

### 4.2. Nitrogen Treatments of Tissue Culture Seedlings

The ‘Daihong’ tissue culture seedlings were cultured on MS medium for 15–20 days after subculture, and then transferred to MS medium containing different concentrations of nitrogen for 0, 6, 12, and 24 h. The plants were frozen in liquid nitrogen and stored at −80 °C until further use.

The nitrogen-deficient culture mediums were prepared as follows: NH_4_NO_3_ and KNO_3_ were removed from MS medium; 0, 4, and 40 mM NO_3_^−^ were added; 40, 3.6, and 0 mM KCl were added, respectively, to supplement the difference in K^+^ concentration; culture mediums with 0, 4, and 40 mM nitrogen concentration were configured. The tissue culture seedlings of ‘Daihong’ were grown in mediums with three nitrogen concentrations for 25 days, and their phenotypic changes were observed. Some of the plants were frozen in liquid nitrogen and stored at −80 °C for the determination of total anthocyanin content, and the remaining were used to determine the nitrogen content.

### 4.3. Extraction and Determination of Total Anthocyanin

The plant material was powdered in liquid nitrogen and 1.0 g of sample powder was added to 10 mL of methanol extraction solution containing 1% HCl. This mixture was used for ultrasonic extraction (frequency, 40 kHz; power, 120 W) at 20 °C for 20 min (KQ-300DE; Kunshan Ultrasonic Instruments Co., Ltd. Kunshan, Jiangsu, China) and then placed at 4 °C for overnight extraction in dark conditions. The extracted solutions were obtained the following day by centrifugation at 10,000× *g* for 10 min at 4 °C (Centrifuge 5804R; Eppendorf, Hamburg, Germany). A 10 mL aliquot of extracting solution was added to the residue and the process was repeated. The two extracted solutions were combined into the test sample, and the anthocyanin contents of various test samples were determined using the pH difference method [59,60].

### 4.4. Nitrogen Measurement

The tissue culture seedlings of ‘Daihong’ were heated at 105 °C for 20–30 min and baked at 70–80 °C for 2–3 days until constant weight. Total nitrogen was determined by micro-Kjeldahl digestion (KDN-102C; Shanghai Xianjian Instruments Co., Ltd., Shanghai, China) following standard methods (China Food and Drug Administration 2016a).

### 4.5. RNA Extraction and Gene Expression Analysis

Gene expression was analyzed by CFX96 Touch Real-Time PCR Detection System (Bio-rad, CFX96 touch). Total RNA was extracted from the leaves, stems, skin, flesh, calli, and tissue culture seedlings of apples using the TIANGEN Plant RNA Extraction Kit (TIANGEN, Beijing, China) according to the manufacturer’s instructions. cDNA was synthesized using the PrimeScript^TM^ II 1st Strand cDNA Synthesis Kit (TaKaRa, Kusatsu, Japan) according to the manufacturer’s instructions. The cDNA was serially diluted 10 times and stored at −20 °C. qRT-PCR was performed using the ChamQ SYBR Color qPCR Master Mix Kit (Vazyme, Nanjing, China) using 1 μL of the template cDNA and 0.5 μL of each gene-specific primer (Supplementary Table S1). The NCBI Primer-BLAST tool was used to design the primers. The *MdActin* gene (XM_029088423.1) was used as an internal reference. The relative expression levels of the target genes were determined using the 2^−ΔΔCT^ method, as described by Livak and Schmittgen [61]. The specificity of each gene was determined using a dissociation curve analysis. All experiments were repeated with three biological replicates and three technical replicates. The gene-specific primer sequences are shown in Supplemental Table S1.

### 4.6. Construction of the CRISPR/Cas9 MdMKK9 Gene Editing Vector and Overexpression Vector

Based on the restriction enzyme sites of the pRI101 vector map, the primers pRI-*MdMKK9*-F and pRI-*MdMKK9*-R (Supplementary Table S1) were designed to amplify the open reading frame region of *MdMKK9* using ‘Daihong’ cDNA as the template. The *MdMKK9* overexpression vector was constructed by connecting *MdMKK9* to the pRI101-AN vector using the *Sal*I and *Bam*HI double restriction sites. The *MdMKK9* overexpression vector was introduced into *Agrobacterium tumefaciens* strain EHA105 and stored at −80 °C.

To examine how the *MdMKK9* gene increases the anthocyanin content of apples under nitrogen stress, we generated a gene mutation of *MdMKK9* through gene editing based on the CRISPR/Cas9 system. The target sequence (TGACGGCGGTGGGAGGGAGGGGG) was selected through the online CRISPR/Cas9 target online predictor (CCTOP) tool [62] using citrus (GCF-00317415.1) as the reference species, and its specificity was verified using the apple database. *MdMKK9* was homologously recombined into the pHDE-35s-Cas9-mCherry-UBQ vector through the *Spe*I restriction site [63]. The MdU6 promoter (accession no. MT584802) was amplified from apple DNA using the primers MdU6-F and MdU6-R (Supplementary Table S1) and was used to promote gRNA expression. The *HygR* gene was replaced with the *KanR* gene to allow convenient selection using kanamycin. The gene editing vector was introduced into *A. tumefaciens* EHA105 and stored at −80 °C for future use.

### 4.7. Agrobacterium-Mediated Transformation of the Calli of ‘Orin’ Apples

The calli of ‘Orin’ apples were used for *Agrobacterium*-mediated genetic transformation [52,64,65]. The calli were subcultured three times at two-week intervals on a subculture medium (MS + 0.4 mg L^−1^ 6-BA + 0.5 mg L^−1^ 2, 4-dichlorophenoxyacetic acid (2, 4-d) pH 5.8) before being used for gene transformation. A 50 μL solution of *A. tumefaciens* EHA105 was added to 50–60 mL of a liquid medium (100 mg L^−1^ Kanamycin + 50 mg L^−1^ Rifampin) and shaken at 28 °C until the bacterial solution concentration was at OD600 = 0.4–0.5. The fresh calli were soaked in an *Agrobacterium* suspension in a suspension buffer (MS + 200 mM Acetosyringone) for 16 min, and then transferred to a selection medium (MS + 0.4 mg L^−1^ 6-BA +0.5 mg L^−1^ 2,4-D + 200 mg L^−1^ Timentin + 100 mg L^−1^ Kanamycin) for transgenic selection. To examine gene mutations and/or overexpression, DNA were extracted from the resistant calli, and the target genes were amplified using PCR. The PCR product was cloned into the pMD19-T simple vector and sequenced. For each antibiotic-resistant calli line, eight clones were randomly picked and sent for sequencing.

### 4.8. Nitrogen Treatments of Transgenic Calli

The calli of wild-type (WT), transformed pRI101-*MdMKK9* (OE) and transformed CRISPR/Cas9-*MdMKK9* (MUT) were placed at 0 mM, 0.4 mM, 4 mM and 40 mM NO_3_^−^ concentration nitrogen MS medium, respectively (the configuration of nitrogen-deficient medium was carried out as described above), cultured in 16 °C incubator for 25 days under dark condition. The calli were snap-frozen in liquid nitrogen after photographing and stored at −80 °C which were used for extraction RNA and anthocyanin.

### 4.9. Statistical Analysis

All data were analyzed with GraphPad Prism 8.0.2 (263) software (GraphPad Software Inc., San Diego, CA, USA). Two-way ANOVA and Tukey’s multiple comparisons test were used to compare the results under different nitrogen concentrations versus the control.

## 5. Conclusions

The excessive use of nitrogen fertilizer in soil is a great waste of energy and seriously affects the global goal of carbon neutralization and carbon peak. Our results suggest that *MdMKK9* plays a role in the adaptation of red-fleshed apple to low nitrogen signals by regulating the anthocyanin accumulation and nitrogen status of fruits. Low nitrogen signal mediated the up-regulated expression of *MdMKK9*, and the expression of *MdMKK9* increased the expression level of anthocyanin synthesis related genes (*MdCHI*, *MdF3H*, *MdANS*, *MdUFGT*) and nitrogen transport gene (*MdNRT2.7*), which promoted anthocyanin synthesis. These results provide new information that can be applied to further investigations into the functions of apple *MKK9* when plants are responding to changes in nitrogen status levels. The interactions between *MdMKK9* and nitrogen transport-related proteins will be the focus of follow-up work, which has potential value for improving the tolerance of apples and other crops (possibly) to nitrogen deficiency.

## Data Availability

The data presented in this study are available in the article and the Supplementary Materials here.

## Supplementary Materials

The following supporting information can be downloaded at: https://www.mdpi.com/article/10.3390/ijms23147755/s1.
