# Supplementary material for: MdMKK9-Mediated the Regulation of Anthocyanin Synthesis in Red-Fleshed Apple in Response to Different Nitrogen Signals
Source: Int J Mol Sci. 2022 Jul 14;23(14):7755. doi: 10.3390/ijms23147755 (PMC9324793; doi:10.3390/ijms23147755)
Supplement: Supplementary file 1 [file ijms-23-07755-s001.zip › ijms-1770884-Supplementary.pdf]

Supplementary Materials

Table S1. The primers used for in the study

Primer Name	ID	Sequence(5′-3′)
		Quantitative real-time PCR primers
MdActin-F	XM_029088423.1	ATGCCAGGGAACATGGTAGA
MdActin-R		TGAGCGAGAAATTGTCAGGG
MdCHS-F	XM_029091251.1	GGAGACAACTGGAGAAGGACTGGAA
MdCHS-R		CGACATTGATACTGGTGTCTTC
MdCHI-F	XM_008394013.3	GGGATA ACCTCGCGGCCAAA
MdCHI-R		GCATCC ATGCCGGA AGCTACAA
MdF3H-F	NM_001293954.1	TGGAAGCTTGTGAGGACTGGGGT
MdF3H-R		CTCCTCCGATGGCAAATCA AAGA
MdDFR-F	NM_001293939.1	GATAGGGTTTGAGTTCAAGTA
MdDFR-R		TCTCCTCAGCAGCCTCAGTTTTCT
MdPAL-F	XM_008357397.3	GAGAGGGAGATCAACTCGGT
MdPAL-R		AACATGAGCTTGCCAATGGA
MdANS-F	OU744958.1	CCAAGTGAAGCGGGTTGTGCT
MdANS-R		CAAAGCAGGCGGACAGGAGTAGC
MdANR-F	OU745007.1	CCTGACAACCACAAGAAG
MdANR-R		GATCACAGCCTGCTATTG
MdUFGT-F	NM_001293991.1	CCACCGCCCTTCCAAACACTCT
MdUFGT-R		CACCCTTATGTTACGCGGCATGT
MdMYB10-F	XM_029107484.1	TGCCTGGACTCGAGAGGAAGACA
MdMYB10-R		CCTGTTTCCCAAAAGCCTGTGAA
MdbHLH3-F	XM_029087752.1	ACC ACCTCAGCCAGAACCT
MdbHLH3-R		CCTTCACCTTGGCTCTTAGTT
MdbHLH33-F	XM_029105700.1	ATGTTTTTGCAACGGAGAGAGCA
MdbHLH33-R		TAGGCGAGTGAACACCATACATTAAAGG
MdWD40-F	XM_008345594.3	AGAATCCCATCTCAGAGCGG
MdWD40-R		AGGGTCGGGTTCGGCTTTATT
MdNFP6.8-F	MG021346.1	ACTCTGCCTATTAGCGTGTTCTTG
MdNFP6.8-R		CATTCCTTTGGGTGACTTGGTTAT
MdNFP6.9-F	XM_008359913.3	CTGGCTGGTCCCACAGTTCTT
MdNFP6.9-R		CTTCATTCCTTTCGGGCACTC
MdNRT2.4-F	XM_008351722.2	CAGAAGGTGAACCCGGAAG
MdNRT2.4-R		CAAGTGGAACGTCCTCATGTG
MdNRT2.7-F	XM_008389221.3	GCAAGCACCTAACAGTCATCTCAC
MdNRT2.7-R		GTTCCCATCAGCACCGACAAT
MdAMT1.5-F	XM_008393058.3	ACAGATAGTGGTAATTATAGGGTGGGT
MdAMT1.5-R		CGTGGTCATGGTACACGTAAGC
MdAMT3.1-F	XM_008395994.3	TCACTGGCCTCGTCTGCATTAC
MdAMT3.1-R		GGTGTCGTCAACTGCGGATAAC
MdMKK2-F	XM_008339518.3	ATCATCCATCGGGACTTC
MdMKK2-R		AATGTATTTGCCTGTTCG
MdMKK3-F	XM_008380986.3	GACCCTTGCGATTCCTAC

MdMKK3-R		CAAACACCCTCATCTCCC
MdMKK4-F	XM_008382039.3	GAGGAACCAGGTCAAGAT
MdMKK4-R		ACATACAAATAGCCCACAT
MdMKK5-F	XM_008344179.3	TAATGTGGGCTATTTGTATG
MdMKK5-R		AAGATTCTGATGGGGTTG
MdMKK6-F	XM_008389149.3	CTCAAGAAGCCAACATCA
MdMKK6-R		ATAGTTTTCCGACCCATT
MdMKK9-F	XM_008376653.3	TCCACTGCCACGCAATCT
MdMKK9-R		GGTGTGGAGGTAGTTGAGGC
		Primers for inducible overexpression of MdMKK9
pRI-*MdMKK9*-F		GTCGACATGGCTCTTATCCGTGAACGCC
pRI-*MdMKK9*-R		GGATCCCCTATCTTTACAGACGAAGGGGTGG
		Primers for construction of Crispr/CAS9 vector
MdU6-pHDE-F		GTCAAACACTGATAGTTTAAACCCGCAAGGAATTTAAGTT
MdMKK9-MdU6-R		CCTCCCTCCCACCGCCGTCAAAGCATTACCTTCAAGTAAG
gRNA-MdMKK9-T-R		GCTATTTCTAGCTCTAAAACCCTCCCTCCCACCGCCGTCA

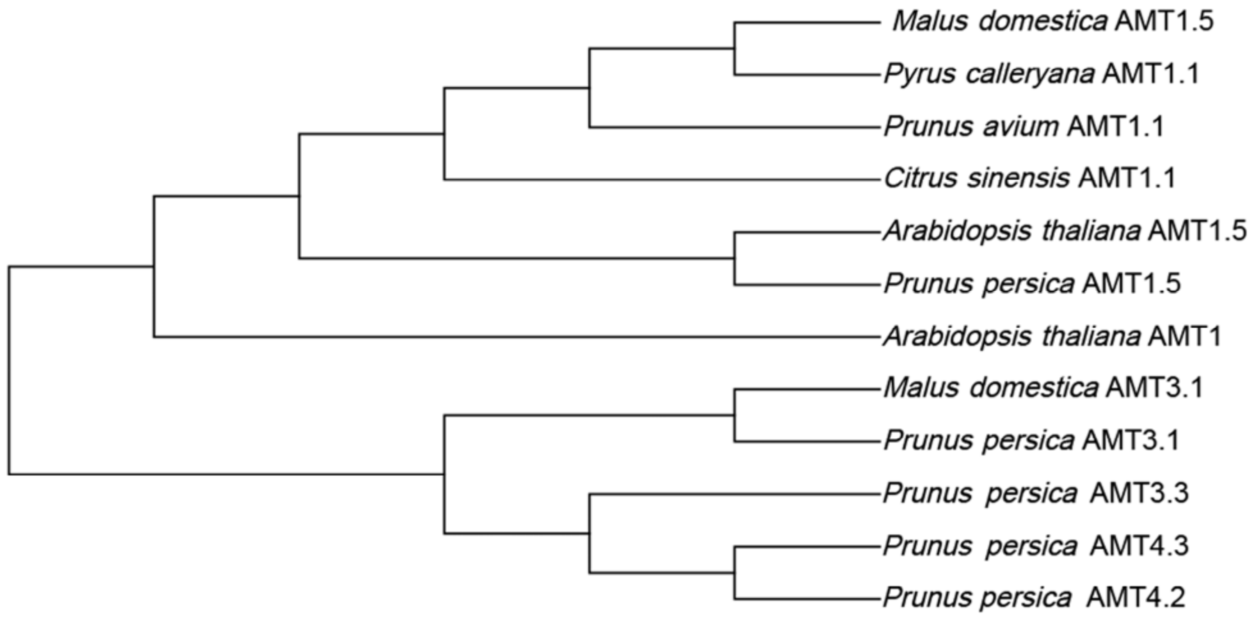

Figure S1. Phylogenetic analysis of MdAMT1.5 and MdAMT3.1 with other plant AMTs.

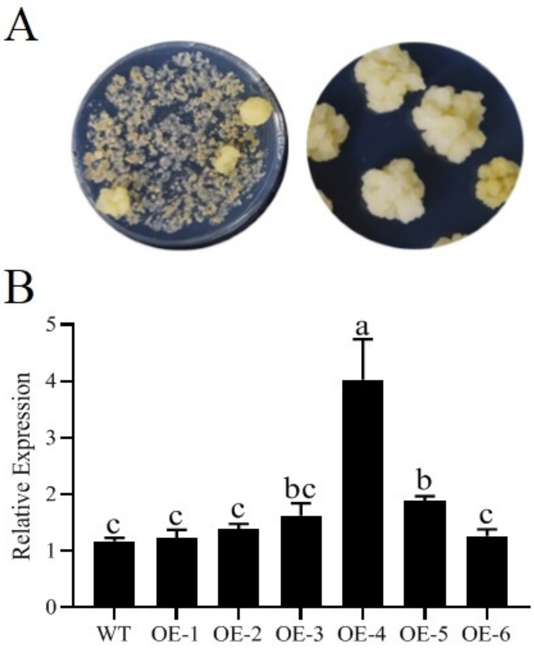

Figure S2. Transformation and identification of *MdMKK9*-overexpressed calli lines (OE). (**A**) Transgenic calli. (**B**) Gene expression of wild-type (WT) 'Orin' calli and 'Orin' calli lines transformed by overexpression of *MdMKK9*. different letters above the bars indicate significant differences ($p < 0.05$). (mean ± SD, n = 4).

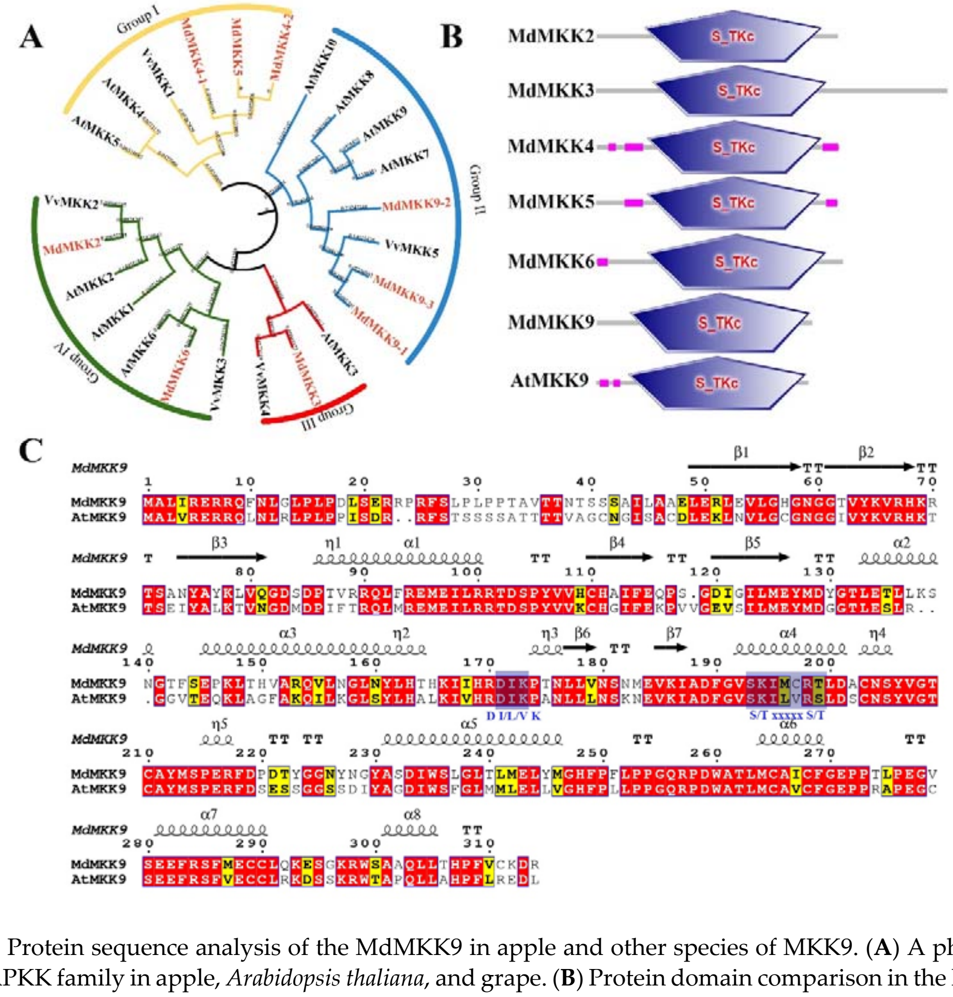

Figure S3. Protein sequence analysis of the MdMKK9 in apple and other species of MKK9. (**A**) A phylogenetic tree of MAPKK family in apple, *Arabidopsis thaliana*, and grape. (**B**) Protein domain comparison in the MdMKK2–MdMKK9 and AtMKK9. (**C**) Sequence alignment of AtMKK9 and MdMKK9. The regions highlighted in blue indicate the S/T xxxxx S/T activation motif and the active site of 'DIK'.

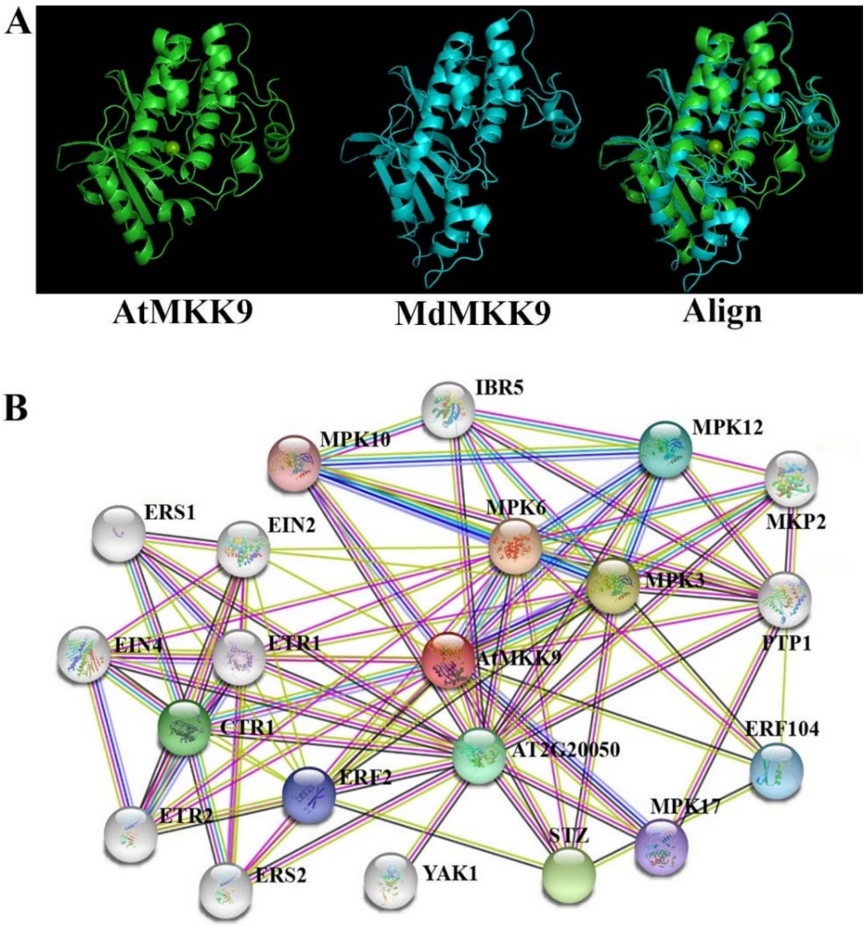

Figure S4. 3D structural comparison of the MdMKK9 and AtMKK9 proteins and a network diagram of AtMKK9-interacting proteins for the functional prediction of MdMKK9. (**A**) 3D protein structures of MdMKK9 and AtMKK9 and their alignment for comparison. (**B**) Network diagram of AtMKK9-interacting proteins for functional prediction. The network has been predicted based on *Arabidopsis thaliana* using the String protein database.
